# Enhanced pathogenicity of low-pathogenic H9N2 avian influenza virus after vaccination with infectious bronchitis live attenuated vaccine

**DOI:** 10.14202/vetworld.2018.977-985

**Published:** 2018-07-24

**Authors:** Zainab Mohamed Ismail, Ayman Hanea EL-Deeb, Mounir Mohamed EL-Safty, Hussein Aly Hussein

**Affiliations:** 1 Department of Virology, Faculty of Veterinary Medicine, Cairo University, Giza, Egypt; 2 Central Laboratory for Evaluation of Veterinary Biologics, Abassia, Egypt

**Keywords:** coinfection, infectious bronchitis virus, low pathogenic H9N2

## Abstract

**Aim:**

In the present study, two experiments were carried out for studying the pathogenicity of H9N2 avian influenza virus (AIV) in broiler chickens after vaccination with different live respiratory viral vaccines.

**Materials and Methods:**

One-day-old specific pathogen-free (SPF) chicks were divided into four groups in each experiment. In experiment 1, Groups 1 and 2 were inoculated with H9N2 AIV through nasal route in 1 day old, Groups 1 and 3 were vaccinated with live infectious bronchitis coronavirus (IBV) vaccine in 5 days old, and Group 4 was left as a negative control. In experiment 2, Groups 5 and 6 were inoculated with AIV subtype H9N2 through nasal route in 1 day old, Group 5 was vaccinated with live IBV vaccine and live Newcastle disease virus (NDV) vaccine in 5 and 18 days old, respectively, Groups 6 and 7 were vaccinated with live NDV vaccine in 18 days old, and Group 8 was left as a negative control. Chicks were kept in isolators for 18 days in the first experiment and 35 days in the second experiment. Tracheal and cloacal swabs were collected from 3, 5, 7, 10, 12, and 15 day’s old chicks from all groups in experiment 1 and 21, 23, 25, and 28 days old from all groups in experiment 2. Quantitative real-time reverse-transcriptase polymerase chain reaction (rRT-PCR) was applied on the collected tracheal swabs for detecting RNA copies of H9N2 AIV. Cloacal swabs and the positive rRT-PCR tracheal swabs were inoculated in 10-day-old SPF embryonated chicken eggs (ECE) to confirm rRT-PCR results. Internal organs (kidney, trachea, and spleen) from all chicken groups were collected weekly for histopathological examination to determine severity of the lesions. Serum samples were collected on a weekly basis for the detection of humoral immune response against H9N2, NDV, and IBV from all chicken groups.

**Results:**

rRT-PCR results with virus titration in ECEs revealed a significant increase in H9N2 AIV titer with extension in the period of viral shedding in Groups 1 and 5. Severe lesion score was observed for Groups 1 and 5. The humoral immune response against H9N2 AIV, NDV, and IBV revealed a significant increase in H9N2 AIV titer in Groups 1 and 5, NDV titer showed a significant increase in Group 7, and IBV titer increased in Groups 1, 3, and 5.

**Conclusion:**

Results demonstrated the increase in pathogenicity of H9N2 AIV, especially when H9N2-infected chicks vaccinated with live IBV vaccine.

## Introduction

Avian influenza (AI) is a respiratory disease in poultry of zoonotic importance caused by influenza A viruses of the family Orthomyxoviridae. Type A influenza viruses are classified into different subtypes according to the two major surface glycoprotein: Hemagglutinin (H) and neuraminidase (N) [1]. Avian influenza virus (AIV) is divided into two groups based on their pathogenicity including highly pathogenic avian influenza (HPAI) in which mortality may be as high as 100%, and low pathogenicity avian influenza (LPAI) which causes milder respiratory disease [2]. Laboratory examinations in specific pathogen-free (SPF) chicken demonstrate that H9N2 AIV is low pathogenic [3]. It is almost one decade that the Middle East and Asian countries are facing frequent outbreaks of H9N2 infection [4,5]. Mixed infection or coinfection of H9N2 AIV with other respiratory pathogens is one of the possible explanations for increasing the economic losses of H9N2 infection in commercial broiler chickens[6,7]. It was reported that infectious bronchitis virus (IBV) infection increased the pathogenicity and extended the period of H9N2 AIV shedding in broiler chickens [6-8].

In Egypt, serological evidence of H9 spread throughout Egypt was recorded on 2009-2010 before isolation of the virus in 2011 [9]. Therefore, there was serological evidence for the presence of H9N2 infection in chicken population during 2001 [10]. In Alexandria, isolated H9N2 virus from chicken flocks suffering from respiratory symptom with high morbidity rates (up to 70%) and mortality rate reaching 15% was analyzed. Blast analysis of the nucleotide sequences from the eight viral genes showed that the recently isolated Egyptian H9N2 strain was closely related to the other Middle East H9N2 strains [11]. The virus shared the common ancestor - the A/Qa/HK/G1/97 isolate - which contributed to the internal genes of the H5N1 virus circulating in Asia [4].

The infections with H9N2 viruses in Egypt are higher in chicken than other species, mostly in apparently healthy broilers, and recorded in layers and breeders [12].

In the present study, two experiments were carried out for studying the pathogenicity of H9N2 avian influenza virus (AIV) in broiler chickens after vaccination with different live respiratory viral vaccines.

## Materials and Methods

### Ethical approval

All applicable institutional guidelines for the care and use of animals were followed in the Central Laboratory for Evaluation of veterinary Biologics (CLEVB).

### AIV

H9N2 AIV strain (A/chicken/EG/1575S/2015) kindly provided by the Central Laboratory for Evaluation of Veterinary Biologics, Egypt, propagated in 10-day-old SPF-ECE for 72 h at 37°C. Allantoic fluids were collected, and EID_50_ was calculated [13]. The collected allantoic fluids with 10^9^ EID_50_/ml were diluted to a final concentration of 10^6^ EID_50_/ml to be used for challenging the chickens.

### Vaccines

Commercial live IBV vaccine (H120 strain, 10^3^ EID_50_/dose) and live Newcastle disease virus (NDV) vaccine (Clone 30 strain, 10^6^ EID_50_/dose) were used in both the experiments. Both were kindly provided by the local producer.

### SPF chicks

131 day-old SPF chicks were obtained from the National SPF egg project, Kom Oshim, EL-Fayoum Governorate, and kept in biosafety isolators; feed and water were kept *ad libitum*. The chicks were divided into eight groups to be used in the experimental trials.

### Experimental design

Two experiments were done for studying the effect of different live respiratory viral vaccines used in field condition on the pathogenicity of H9N2 AIV.

### Experiment 1

The SPF broiler chicks were divided into four groups numbered 1-4. The chicks were raised for 18 days in isolators. H9N2 AIV was inoculated through nasal route; live IBV vaccine was administrated in drinking water by the recommended dose of the producer (Table-1).

**Table-1:** The different treatment used in experiment 1.

Groups/No	Number of birds	1 day old	5 days old
1	20	H9N2	IBV vaccine
2	20	H9N2	-
3	15	-	IBV vaccine
4	10	-	-

IBV=Infectious bronchitis coronavirus

### Experiment 2

The SPF broiler chicks were divided into four groups numbered 5-8. The chicks were raised for 35 days in isolators. H9N2 AIV was inoculated through nasal route; live IBV and NDV vaccines were administrated in drinking water by the recommended dose of the producer (Table-2).

**Table-2:** The different treatment used in experiment 2.

Groups/No	Number of birds	1 day old	5 days old	18 days old
5	20	H9N2	IBV vaccine	NDV vaccine
6	20	H9N2	-	NDV vaccine
7	15	-	-	NDV vaccine
8	10	-	-	-

IBV=Infectious bronchitis coronavirus, NDV=Newcastle disease virus

### Serology

Sera were collected weekly in each group in the two experiments and tested by hemagglutination inhibition (HI) test for the detection of antibodies against H9N2 AIV and NDV [14,15] and tested by ELISA for the detection of antibodies against infectious bronchitis virus using Infectious Bronchitis Virus Antibody Test Kit (Jovac).

The interpretation of ELISA results was as follows:

ELISA unit (EU) calculation:

Ratio sample/positive (S/P) = (Abs_test sample_−Abs_Negative_)/(Abs_positive_−Abs_negative_), EU_sample_=(S/P)×100.

### Organ histopathology

Internal organs (kidney, trachea, and spleen) were collected weekly in each group in the two experiments and were fixed in 10% formol saline embedded in paraffin, sectioned at 4 µm thickness, and then stained with hematoxylin and eosin [16]. The obtained slides were examined by the light microscope. Pathological scores were recorded for each chicken group, and a comparative study was applied.

### Virus shedding

Tracheal and cloacal swabs were collected from 3, 5, 7, 10, 12, and 15 days old from all groups in experiment 1. Swabs were collected from 21, 23, 25, and 28 days old from all groups in experiment 2.

### Real-Time reverse-transcriptase-polymerase chain reaction (rRT-PCR)

RNA extraction was completed using QIAamp Viral RNA Mini Kit (QIAGEN) catalog No.52904. Real-time RT-PCR was applied using Quantitect probe, catalog No. 204443 and was used for the quantification of H9N2 AIV titers in tracheal swabs using the specific primers and probe as mentioned in Table-3.

**Table-3:** Oligonucleotide primers and probes used in real-time PCR.

Virus	Gene	Primer/probe sequence 5’-3’	Reference
H9	H	H9F GGAAGAATTAATTATTATTGGTCGGTAC	[54]
		H9R GCCACCTTTTTCAGTCTGACATT	
		H9 Probe [FAM] AACCAGGCCAGACATTGCGAGTAAGATCC[TAMRA]	

PCR=Polymerase chain reaction

### Confirmation of positive rRT-PCR tracheal swabs by inoculation in specific pathogen-free embryonating chicken eggs (SPF-ECE)

Positive tracheal swabs by rRT-PCR were inoculated in SPF-ECE for virus titration. Furthermore, cloacal swabs collected from different chicken groups were inoculated for virus detection and titration [17].

### Statistical analysis

Data were expressed as mean±standard deviation. Statistical comparison between the mean of the different groups was made by one-way ANOVA. Multiple comparison between groups (*post hoc*) and least significant degree were made using SPSS version [18]. A p≤0.05 was assumed for statistical significance. Charts were made by GraphPad Prism 7.

## Results

### Serological findings

#### Serum HI titer of H9N2 AIV

**Figure-1:**
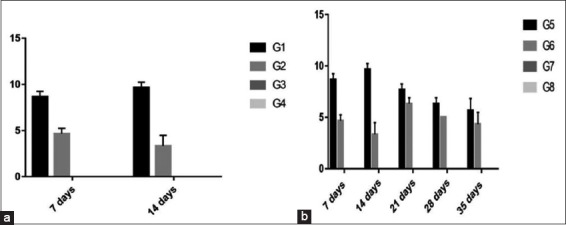
Column chart showing serum hemagglutination inhibition titer of H9N2 avian influenza virus (expressed as log 2). (a) Experiment 1, (b) Experiment 2.

HI activity which is an indication of virus replication showed that:

- In experiment 1, there was a significant increase in Group 1 (8.7 and 9.6) than Group 2 (4.6 and 3.3) at 7 and 14 days, respectively. Sera were ≤ 2 in Groups 3 and 4.
- In experiment 2, there was a significant increase in Group 5 (8.7, 9.6, 7.6, 6.3, and 5.6) than Group 6 (4.6, 3.3, 6.3, 5, and 4.3) at 7, 14, 21, 28, and 35 days, respectively. Sera were ≤ 2 in Groups 7 and 8 (Figure-1).

#### Serum HI titer of NDV

**Figure-2:**
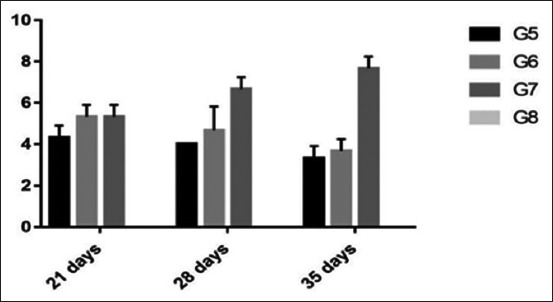
Column chart showing serum hemagglutination inhibition titer of Newcastle disease virus (expressed as log 2).

The serology of NDV in experiment 2 showed a significant increase at 28 and 35 days in Group 7 (6.7 and 7.7) than other Groups 5 (4 and 3.3) and 6 (4.7 and 3.6), respectively. There was decreasing NDV antibody titer in both the Groups 5 and 6 by increasing the age. All tested samples in group 8 were ≤ 2 (Figure-2).

#### Serum ELISA titer of IBV

**Figure-3:**
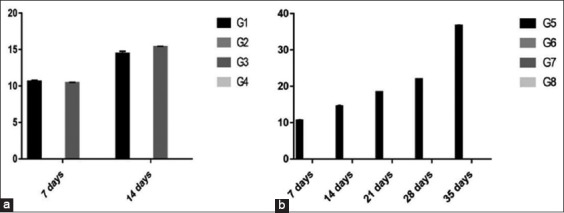
Column chart showing serum ELISA titer of infectious bronchitis coronavirus (expressed as ELISA units). (a) Experiment 1, (b) Experiment 2.

The serology of IBV revealed that:

- In experiment 1, there was a significant increase in ELISA units in Group 3 (15.4) than Group 1(14.4) at 14 days old, while the same results obtained at 7 days old. All the tested samples in Groups 2 and 4 were ≤10.
- In experiment 2, increasing IBV antibody titer by increasing the age in Group 5 besides ≤10 ELISA units in all remaining groups (Figure-3).

### Histopathology

#### Histopathological results of experiment 1

Histopathological examination of the organs collected from group 1 revealed severe denudation of renal tubules and mul­tifocal histiocytes infiltration and focal mononuclear cell infiltration in kidney, severe focal hyperplasia of the tracheal lining epithelium with activation of mucous glands, also the muscular layer showed hemorrhage in trachea and severe depletion of lymphocytes and severe focal coagulative necrosis in spleen. On the other hand, examination of the organs collected from group 2 revealed only mild congested blood vessels in kidney, mild hyperplasia of lining epithelium in trachea and mild thickening of the capsule in spleen. Organs collected from Group 3 results showed sloughed epithelium in the lumen in tra­chea. Meanwhile, organs collected from group 4 showed no changes (Table-4).

**Table-4:** The score of pathological lesions for all groups in experiment 1.

Organ	Group	Age of birds					

		7 days			14 days		
	
		S1	S2	S3	S1	S2	S3
Kidney	1	+++	++	+++	+++	++	+++
	2	+	-	+	+	+	-
	3	-	-	-	-	-	-
	4	-	-	-	-	-	-
Trachea	1	++	++	++	++	+++	++
	2	-	-	-	+	+	+
	3	+	+	-	-	-	-
	4	-	-	-	-	-	-
Spleen	1	+	++	+	+++	+++	+++
	2	+	+	+	+	-	+
	3	-	-	-	-	-	-
	4	-	-	-	-	-	-

S1, S2, and S3: Number of tissue samples examined. (+): Mild/(++): Moderated/(+++): Severe

#### Histopathological results of experiment 2

Histopathological studies in experiment 2 revealed severe denudation and focal infiltration with mononuclear cells; intertubular congestion and intertubular hemorrhage in kidney, severe activation of mucous glands, hyperplasia of lining epithelium with sloughed epithelium and submucosal hemor­rhage in trachea and severe focal hemorrhage and lymphoblasts infiltration, thickening of the wall of blood vessels with focal depletion and thickening of the capsule in spleen in group 5. Contrarily, group 6 Histopathological results showed only flattening and degeneration of some renal tubules with cystic formation and mild congested blood ves­sels in kidney, mild focal hyperplasia of the lining epithelium with submucosal edema and sloughed epithelium in the lumen in trachea. Moreover, thick­ening of the wall of blood vessels with perivascular edema and mildly congested blood vessels in spleen; organs from group 7 and group 8 showed no changes (Table-5).

**Table-5:** The score pathological lesions for all groups in experiment 2.

Age of birds	Group	Organ								

		Kidney			Trachea			Spleen		
		
		S1	S2	S3	S1	S2	S3	S1	S2	S3
7 days	5	+++	++	++	++	++	++	++	++	++
	6	+	-	+	-	-	-	+	+	+
	7	-	-	-	-	-	-	-	-	-
	8	-	-	-	-	-	-	-	-	-
14 days	5	+++	++	+++	++	+++	++	+++	+++	++
	6	+	+	-	+	+	+	+	-	+
	7	-	-	-	-	-	-	-	-	-
	8	-	-	-	-	-	-	-	-	-
21 days	5	+++	++	++	++	++	+	+++	++	+++
	6	++	+	+	+	++	+	+	+	++
	7	-	-	-	-	-	-	-	-	-
	8	-	-	-	-	-	-	-	-	-
28 days	5	++	+	+	++	+	+	++	+	++
	6	+	-	+	+	-	+	-	+	+
	7	-	-	-	-	-	-	-	-	-
	8	-	-	-	-	-	-	-	-	-
35 days	5	++	++	++	++	+	++	++	+	+
	6	++	+	++	+	+	+	+	+	+
	7	-	-	-	-	-	-	-	-	-
	8	-	-	-	-	-	-	-	-	-

### Virus shedding

#### Real-Time RT-PCR

**Figure-4:**
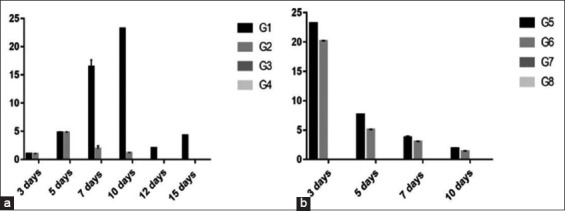
Column chart showing RNA copies of H9 using rRT-polymerase chain reaction in chicken groups and different time conditions. (a) Experiment 1, (b) Experiment 2.

Results of H9N2 AIV shedding using rRT-PCR assay showed that:

- In experiment 1, there was a significant increase in RNA copies in Group 1 (1.65×10 [ct 28.07] and 2.319×10 [ct 27.58]) than Group 2 (1.89 [ct 30.73] and 1.19 [ct 31.91]) observed clearly at 7 and 10 days, respectively. Virus shedding extended in Group 1 up to 15 days versus Group 2 only up to 10 days.
- In experiment 2, there was a significant increase in RNA copies in Group 5 with copies ranged between 2.319×10 (ct 27.80) at 21 days and 1.86 (ct 31.16) at 28 days than Group 6 with copies ranged between 2.017×10 (ct 27.81) at 21 days and 1.36 (ct 31.68) at 28 days (Figure-4).

### Titration of H9N2 AIV in SPF-ECE

#### Virus titration in egg from tracheal swabs

**Figure-5:**
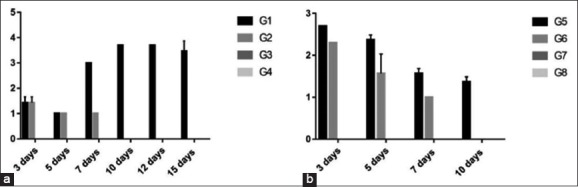
Column chart showing virus titration in egg from tracheal swabs (expressed as log 10) in different groups and different time conditions. (a) Experiment 1, (b) Experiment 2.

Titration of H9N2 AIV from tracheal swabs revealed that:

- In experiment 1, there was a significant increase in Group 1 (3) than Group 2 (1) observed clearly at 7 days old with virus shedding extended in Group 1 up to 15 days versus in Group 2 only up to 7 days.
- In experiment 2, there was a significant increase in Group 5 (2.7, 2.3, and 1.5) than Group 6 (2.3, 1.5, and 1) at 3, 5, and 7 days postvaccination, respectively, with virus shedding extended in Group 5 up to 10 days versus Group 6 only up to 7 days (Figure-5).

#### Virus titration in egg from cloacal swabs

**Figure-6:**
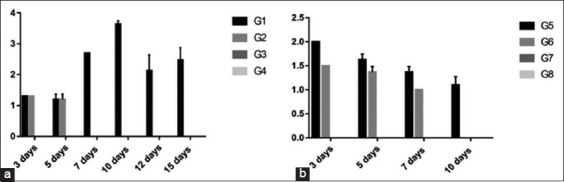
Column chart showing virus titration in egg (expressed as log 10) from cloacal swabs in different groups and different time conditions. (a) experiment 1, (b) experiment 2.

H9N2 AIV titration from cloacal swabs revealed that:

- In experiment 1, there was a significant increase in virus shedding in Group 1 extended up to 15 days versus Group 2 only up to 5 days.
- In experiment 2, there was a significant increase in Group 5 (2, 1.6, and 1.4) than Group 6 (1.5, 1.3 and 1) at 3, 5, and 7 days old with virus shedding extended up to 10 days in Group 5 versus Group 6 only up to 7 days (Figure-6).

## Discussion

H9N2 AIV causes great economic losses in broiler chickens although the virus is classified as low pathogenic virus [4,13,18,19] and manifests mild clinical signs and respiratory disease with low mortality not more than 5% [20]. Mixed infection with other respiratory pathogens as IBV circulating in the field may be one of the causes for increasing pathogenicity of the virus causing great losses in poultry production [21]. Furthermore, it was reported that mixed infection of AIV H9N2 with *Staphylococcus aureus* and *Haemophilus paragallinarum* activated HA of the virus and increased its replication and spread [22]. Furthermore, *Escherichia coli* has a vital role in viral spread and virulence of H9N2 AIV [23].

On the other hand, it is well known that the hemagglutinin glycoprotein for influenza viruses is produced as a precursor, HA0, which requires cleavage by host proteases before it is functional and virus particles are infectious [24]. The HA0 precursor proteins of AIVs of low virulence for poultry have a single arginine at the cleavage site and another at position −4. These viruses are restricted to cleavage by host proteases such as trypsin-like enzymes and thus limited to replication at sites in the host where such enzymes are found, i.e., the respiratory and intestinal tracts[25,26]. HPAI viruses have multiple basic amino acids (arginine and lysine) at their HA0 cleavage sites [1,27,28] and appear to be cleavable by ubiquitous proteases. These viruses are able to replicate and spread throughout the bird, damaging vital organs and tissues which results in disease and death [24]. Indeed, trypsin-like proteases are essential for the cleavage activation of the HA and so play a vital role in viral pathogenicity [29,30].

In the present study, lesions of internal organs (kidney, trachea, and spleen) of the group coinfected with H9N2 AIV and live IBV vaccines were significantly higher than the H9N2 AIV-infected group in experiment 1; histopathological changes in the kidney were as the results of direct influence of H9N2 AIV replication in renal tubules [31-33]. There are several reports on kidney lesions in chickens infected with low pathogenic AIVs [31,32,34]. Focal and diffuse interstitial lymphocyte infiltration observed and indicated that the kidney has the potential for survival of the virus. Significant changes were observed in spleen, and according to Swayne and Slemons [35], such changes in the spleen could be as the result of the host immune response to H9N2 AIV. Trachea showed prominent severe lesions, and similar findings were observed by others where more cell injuries in the tissues of respiratory system and GIT were recorded [36]. The presence of trypsin-like enzyme may be the cause of cleavage of the HA protein of LP AIV and the coinfection of the virus with live IBV vaccine indicates that live IBV vaccine increases the replication of H9N2 AIV and its spread in the tissues [8].

Taken together, results of rRT-PCR assay and virus titration revealed that the vaccination with live IBV vaccine postinfection of the chicks with H9N2 AIV has a great effect on enhancement of the virulence of H9N2 AIV, extend the period of viral shedding, and increase the pathogenicity versus H9N2 AIV-infected group in the experiment [7,8]. Such observation may explain the great economic losses observed in the field due to the presence of both viruses in the infected chickens.

It has been demonstrated that live IBV vaccine, H120 strain, exacerbates the manifestation of experimental H9N2 AIV infection in broiler chickens [37,38] as IBV could provoke ciliostasis in the host ciliated airways [39] and therefore facilitate the opportunity for other related pathogens to induce their pathogenicity. Furthermore, live IBV vaccine coinfection may have provided the protease enzymes and enhanced H9N2 AIV pathogenicity in this study as it has been reported that a trypsin-like serine protease domain is encoded by coronavirus IBV [40,41]. Furthermore, it has been established that stimulation of host cells to produce or secrete more protease and the destruction of endogenous cell protease inhibitors may increase trypsin-like protease activity and enhanced influenza virus pathogenicity [42].

In the present study, the results of experiment 1 revealed that HI titer of H9N2 AIV increased in the group infected with H9N2 AIV and vaccinated with live IBV vaccine versus H9N2 AIV-infected group, and these results indicated that IBV could enhance the propagation of H9N2AIV and consequently an increase in H9N2 AIV HI titer [4,8]. Furthermore, the serology of IBV increased when combined with H9N2 AIV, and this explained the synergistic effect of coinfection between two viruses which was previously reported [8].

The results of HI titer of H9N2 AIV in experiment 2 revealed that H9N2 AIV HI titer was reduced in the group infected with H9N2AIV and vaccinated with live NDV vaccine versus the group infected with H9N2 AIV and vaccinated with both live IBV and NDV vaccines, indicating that live NDV vaccine interfered with H9N2 AIV replication and this is in accordance with others [43-45]. Interference between live NDV vaccine and H9N2 AIV explained the reduction of NDV titer in Groups 5 and 6 in the second experiment while NDV titer was significantly higher in Group 7.

Histopathological studies in experiment 2 revealed that the infection with H9N2 AIV in birds vaccinated with live NDV alone induces mild lesions. Meanwhile, infection with H9N2 AIV in birds vaccinated with both live IBV and NDV vaccines induced severe lesions in all collected internal organs and these results indicated that live NDV vaccine has no significant effect on the severity of H9N2 AIV [46]. Results of rRT-PCR with virus titration in egg in experiment 2 revealed that in the group infected with H9N2 AIV and vaccinated with live NDV vaccine, virus shedding was interfered when compared with the group infected with H9N2 AIV and vaccinated with both live IBV and NDV vaccines. These results are in consistent with the previous reports [45] which stated that coinfection of LPAIV and NDV resulted in low number of cloacal swabs in which both viruses were detected; therefore, coinfection affected the replication dynamics of these viruses. The results are also consistent with other reports [47] which demonstrated that chicken infected with LPAIV and coinfected with NDV showed mild clinical signs and moderate inflammation in the epithelia of the nasal trachea and air sacs, and the LPAIV shedding patterns were affected in chickens exposed to LPAIV and NDV.

Based on the obtained results, there was no effect of live NDV vaccine on the severity of lesions caused due to H9N2 AIV.Coinfections of H9N2 AIV and NDV have been previously studied *in vitro* using cell cultures or chicken embryos, and interference between these viruses has been reported, with one virus inhibiting the growth of the other [48-50]. Viral interference is a phenomenon in which a cell infected by a virus does not allow multiplication of a second virus [51]. Viral interference can be explained by different mechanisms including competing by attachment interference, therefore, reducing or blocking of receptor sites for the super-infecting virus; competing intracellularly for replication host machinery; and virus-induced interferon interference [52]. NDV and H9N2 AIV replicate in cells where there are trypsin-like enzymes such as in the upper respiratory and intestinal epithelia and might compete for the same target cells or replicate in adjacent cells [53].

## Conclusion

Collectively, the overall results of the present study demonstrated that live IBV vaccine postinfection with AIV subtype H9N2 increases its pathogenicity and this may be one of the factors causing economic losses observed in the past 5 years in broiler chickens in Egypt.

## Authors’ Contributions

ZMI conducted the experiment and drafted the manuscript, HAH and AHE designed and followed up the experiment and critically reviewed the manuscript, and MME participated in designing and followed up the practical work. This work is part of the master degree of ZI supervised by HAH, A EL-Deeb, and M EL-Safty. All authors read and approved the final manuscript.
